# Optimization and Characterization of Crosslinked Chitosan-Based Oleogels Based on Mechanical Properties of Conventional Solid Fats

**DOI:** 10.3390/polym17111526

**Published:** 2025-05-29

**Authors:** Gabriela Baptista Brito, Jorge da Silva Pinho-Jr, André da Silva Guimarães, Carlos Adam Conte-Júnior, Marcio Nele, Daniel Perrone, Vanessa Naciuk Castelo-Branco

**Affiliations:** 1 Instituto de Química, Universidade Federal do Rio de Janeiro, CT, Bloco A, Cidade Universitária, Av. Athos da Silveira Ramos 149, Rio de Janeiro 21941-909, Brazil; bbrito.gabriela@gmail.com (G.B.B.);; 2 Faculdade de Farmácia, Universidade Federal Fluminense, Rua Dr. Mário Viana, 523, Niteroi 24241-000, Brazil; 3 Programa de Engenharia Química, Universidade Federal do Rio de Janeiro, CT, Bloco G, Cidade Universitária, Av. Horácio Macedo 2030, Rio de Janeiro 21941-914, Brazil

**Keywords:** emulsion-template, fat replacer, organogel, trans fatty acids

## Abstract

Industrial *trans* and saturated fatty acids, which are key components of solid fats used in food products, should be replaced with unsaturated fatty acids from vegetable oils to reduce cardiovascular risk. However, unsaturated oils lack the structured networks required to replicate the technological properties of solid fats. Oleogelation, especially using polymer-based networks, offers a promising solution. This study optimized chitosan-based oleogels crosslinked with vanillin to mimic the texture of butter, partially hydrogenated fat, margarine, and palm fat while minimizing oil loss. Oleogels were prepared via the emulsion-template method and optimized through a central composite design combined with a desirability function, evaluating the effects of chitosan, vanillin, Tween^®^ 60 concentrations, oil type (canola or soybean), and storage temperature (4 °C or 25 °C). Optimized oleogels were characterized for their rheological and microstructural properties. Chitosan concentration primarily governed oil loss, hardness, and adhesiveness of oleogels, independent of the oil phase and storage temperature. However, storage at 4 °C reduced oil loss but increased the hardness and adhesiveness compared to storage at 25 °C. The highest desirability scores (0.72 to 0.94) were achieved in soybean oil oleogels with 0.99% chitosan, 0.24–0.32% vanillin, and 0.17–0.18% Tween^®^ 60, closely mimicking the texture of butter and margarine. These oleogels demonstrated stronger networks, enhanced gel strength, and elasticity, positioning them as viable alternatives to conventional solid fats.

## 1. Introduction

The sensory and structural attributes of food products are critical factors in consumer acceptance, as they influence quality perception and overall eating experience. Solid fats play a key role in defining the structure, rheological behavior, and sensory properties of food systems. These functionalities depend on the composition and distribution of fatty acids in triacylglycerols, particularly industrially produced trans and saturated fatty acids, which govern the melting behavior, crystallization patterns, and overall functionality of fats in food matrices [1,2]. However, excessive consumption of these fatty acids has been associated with adverse health effects, including an increased risk of cardiovascular diseases, driving efforts to replace them with unsaturated fatty acids from vegetable oils, which offer health benefits [3]. Nevertheless, unsaturated fatty acids lack the ability to form structured networks like saturated and trans fatty acids, compromising the sensory and mechanical performance of food products. To address this challenge, the conversion of liquid oils into gel-like structures, such as oleogels, has emerged as a promising strategy to improve the fatty acid profile of food products, while retaining desirable technological properties [4,5].

Oleogels consist of an oil phase entrapped within a self-assembled, polymeric, or crystalline three-dimensional network formed by one or more structuring agents (i.e., oleogelators) that can mimic the textural and mechanical functionality of solid fats while preserving the nutritional properties of vegetable oils containing unsaturated fatty acids and natural antioxidants [4]. Among the different types of oleogels, those structured by biopolymers—such as chitosan, a renewable and cost-effective food additive—have garnered significant interest [6,7].

Chitosan, a versatile biopolymer, combines renewable sourcing with multifuncional properties, including its renewable origin, antioxidant, antimicrobial, and anti-inflammatory activities. It also acts as a fat absorption inhibitor, immunomodulator, and antitumor agent [8]. Its effectiveness as an oil-structuring agent stems from its ability to form gels at low concentrations (<2%), especially when combined with crosslinking agents that improve its solubility in oil [6,9,10,11,12]. Vanillin, for instance, chemically modifies the amino groups (NH_2_^+^) of chitosan via crosslinking with its aromatic aldehyde group, forming Schiff bases. This reduces the electrostatic repulsion between polycationic chitosan chains, enabling the formation of a stable three-dimensional network that retains oil [6,11,12]. Previous studies on chitosan-based oleogels crosslinked with vanillin—using canola oil [6], extra-virgin olive oil [13], or camellia oil bodies [11,12] prepared via the emulsion-template method—have revealed variations in thermal stability and mechanical and physical properties. These differences are attributed to factors such as oil phase type, chitosan-to-vanillin ratio, interactions with emulsifiers like Tween^®^ 60, and processing or storage conditions.

Despite these findings, no systematic evaluation has explored how these factors interact or how they can be optimized to enhance the mechanical and structural properties of chitosan-based oleogels. A comprehensive understanding of these interactions, particularly through multivariate statistical approaches, is essential for rationally designing oleogels that replicate the characteristics of butter, partially hydrogenated fats, margarine, and palm fat. This would expand their versatility and applicability across diverse food products.

Therefore, the present study investigated the effects of chitosan, vanillin, and Tween^®^ 60 concentrations, oil phase type, and storage temperature on the textural properties and oil loss of chitosan-based oleogels crosslinked with vanillin. Multiple response optimization using the desirability function was conducted to identify formulations with hardness and adhesiveness comparable to butter, partially hydrogenated fats, margarine, and palm fat, while minimizing oil loss. Optimized oleogels were further characterized for rheological and microstructural properties to assess their potential as solid fat replacers. At the end of this study, it was found that those oleogels prepared with soybean oil showed superior textural characteristics, closely resembling the properties of traditional solid fats like butter and margarine. Overall, these oleogels exhibited enhanced structural strength and elasticity, highlighting their potential as viable, healthier alternatives for replacing conventional solid fats in food applications.

## 2. Materials and Methods

### 2.1. Samples, Reagents, and Solvents

Commercial refined canola and soybean oils and commercial solid fats (butter, margarine from brands #1 and #2, partially hydrogenated fat from brands #1 and #2, and palm fat) were purchased at local markets (Rio de Janeiro, Brazil) between 2019 and 2022. The lipid content of these samples as reported by the manufacturers is detailed in Table S1. Chitosan (75–85% deacetylation degree, molecular weight range of 190–310 kDa; viscosity range of 200–800 cP), 3-methoxy-4-hydroxybenzaldehyde (vanillin, 99% purity, Generally Recognized as Safe (GRAS) under 21 CFR 182.60 (FDA, 2021)), and polyethylene glycol sorbitan monostearate (Tween^®^ 60, GRAS under 21 CFR 172.836 (FDA, 2021)) were purchased from Sigma-Aldrich (São Paulo, Brazil). The FTIR spectrum of chitosan used in the present study is presented in Figure S1. Ethanol and glacial acetic acid were of analytical grade (Biograde Chemicals, Goiânia, Brazil).

### 2.2. Chitosan-Based Oleogel Preparation

Chitosan-based oleogels were prepared by the emulsion-templated approach using vanillin as a crosslinking agent and Tween^®^ 60 as the emulsifier, following the method described by [6]. Canola or soybean oil were used as the oil phase. The concentrations of chitosan, vanillin, and Tween^®^ 60 in the final oleogels were previously determined by the experimental design described in Section 2.3. All oleogels were stored at 4 °C for at least 48 h before analysis.

### 2.3. Experimental Design

To evaluate the effects of the concentration of chitosan, vanillin, and Tween^®^ 60 on the oil loss, hardness, and adhesiveness of chitosan-based oleogels, a face-centered central composite design—comprising a three-level, three-factor full factorial design with three central points—was used, resulting in 17 randomized experimental runs. The design matrix (Table 1) was prepared using Statistica software (version 8.0, StatSoft Inc., Tulsa, OK, USA), with the minimum (coded as −1), central (coded as 0), and maximum (coded as +1) levels for each independent variable, as follows: chitosan (0.42%, 0.75%, 1.07%), vanillin (0.05%, 0.52%, 1.00%), and Tween^®^ 60 (0.00%, 0.25%, 0.50%). The concentration ranges of chitosan, vanillin, and Tween^®^ 60 were selected based on previous studies conducted by our research group [6] and supported by findings in the literature [11], with the aim of identifying an optimal balance among these components. Furthermore, economic considerations were factored into the selection process, as we aimed to minimize the use of chitosan and vanillin while still achieving the desired technological performance, thereby contributing to the cost-effectiveness of the oleogel formulations. All runs were performed in duplicate. The response variables were analyzed after 24 h of storage at 4 °C or 25 °C, resulting in four experimental designs: (1) chitosan-based oleogels made with canola oil and stored at 4 °C for 24 h; (2) chitosan-based oleogels made with canola oil and stored at 25 °C for 24 h; (3) chitosan-based oleogels made with soybean oil and stored at 4 °C for 24 h; and (4) chitosan-based oleogel made with soybean oil and stored at 25 °C for 24 h.

A second-order polynomial model was fitted by the response surface methodology (RSM) to determine the relative contributions of chitosan, vanillin, and Tween^®^ 60 concentrations and their interactions on the oil loss, hardness, and adhesiveness of oleogels. The quality and significance of fitting models was tested with analysis of variance (ANOVA). The best-fit surface models were selected based on the coefficient of determination (R^2^), adjusted coefficient of determination (adj-R^2^), and agreement between experimental and predicted values for each response variable, assessed using the paired Student’s *t*-test. Terms with *p*-values > 0.05 were removed from the models.

### 2.4. Analysis of Response Variables: Oil Loss, Hardness, and Adhesiveness of Oleogels

Oil loss was determined gravimetrically as the percentage of oil exuded naturally after the oleogels were stored for 24 h at 4 °C or 25 °C, following the method described by [14]. Hardness and adhesiveness were measured using the texture profile analysis (TPA) protocol with a TA-XT plus Texture Analyzer (Stable Micro Systems, Surrey, UK) equipped with a 5 kg load cell and a cylindrical probe (36 mm in diameter). Oleogel samples (40 g) were placed in a 100 mL beaker (height × internal diameter: 75 × 50 mm) and subjected to a double-compression test (pre-test and post-test speeds of 13 mm/s, test speed of 10 mm/s). The trigger force was set at 0.1 N, with a 5 s interval between compressions [6]. All texture analyses were conducted in duplicate after oleogels were stored at 4 °C or 25 °C for 24 h. TPA of the commercial solid fats was measured under the same storage conditions of chitosan-based oleogels.

### 2.5. Multiresponse Optimization by Desirability Function and Verification of Models

The desirability function was employed to optimize oleogel formulations that mimic the hardness and adhesiveness of solid fats while minimizing oil loss, based on RSM-generated models. This optimization was applied to oleogels prepared with canola oil or soybean oil and stored at 25 °C for 24 h. Optimization was performed by setting the target values of hardness and adhesiveness to match those of commercial solid fats (butter, margarine brands #1 and #2, hydrogenated fat brands #1 and #2, and palm fat) while setting oil loss targets to the minimum observed in the experimental range. The desirability function transforms each response into a value ranging between 0 and 1, where 1 represents the optimal value and 0 indicates the least desirable condition. The individual desirability functions were combined to identify the optimal condition across all the responses, termed the global desirability function (D). This analysis was performed using Statistica software (version 8.0, StatSoft Inc., Tulsa, OK, USA). The optimal oleogel formulations for each solid fat were tested in independent experiments to verify the predictability of the models and further analyzed for their microstructure and rheological properties.

### 2.6. Determination of Microstructure and Rheological Properties of Optimized Oleogels

#### 2.6.1. Microstructure of Oleogels

The microstructures of optimized oleogels and their corresponding emulsions and dried products were assessed using an upright microscope, AxioImager A2.m (Carl Zeiss, Jena, Germany), equipped with a 5.0-megapixel MRC5 AxioCam and Axio Vision 4.8 software (Carl Zeiss, Germany). A single drop of emulsion or small slices of dried emulsions/oleogels were placed on a glass microscope slide, covered with a coverslip to form a thin film, and visualized under bright-field and polarized light. The polarized light technique was employed, and a 100× objective lens (1000× magnification) was used for visualizing the emulsions, and a 20× objective lens (200× magnification) was used for visualizing dried emulsions and oleogels. Samples were equilibrated at 25 °C on a hot stage connected to a Linkam T95-PE System Controller (Linkam Scientific Instrument Ltd., Surrey, UK).

#### 2.6.2. Rheological Measurements

Oscillatory and rotational tests of the optimized oleogels were performed using a controlled stress rheometer (Discovery HR-3, TA Instruments, New Castle, DE, USA) with a Peltier temperature control system and TRIOS software (version 5.1.1, Lite) following the methodology described by [6]. A parallel plate system with an upper crosshatched geometry with a diameter of 60 mm was utilized, and a gap of 1000 µm was established for the tests. Oscillatory amplitude strain sweep tests (frequency: 1.0 Hz; strain: 0.01 to 100.0%) were performed to determine the values of the storage modulus (G′), loss modulus (G″), critical stress (σ critical), critical strain (γ critical), yield stress (σ yield), and yield strain (γ yield). The linear viscoelastic region (LVR) was defined as the range where G′ deviated by no more than 5% from its plateau value. Frequency sweeps (0.1 Hz to 10.0 Hz) and temperature ramps (5 °C to ↔ 80 °C, at 5 °C/min, and 1 Hz) were measured within the LVR and performed at 0.1% strain. Rotational flow measurements (shear rates: 1 to 100 s^−1^) were used to analyze viscosity. The structure-recovery properties were evaluated by a thixotropic test, applying alternating strain in three 300 s intervals: 0.1% (interval 1), 10% (interval 2), and 0.1% (interval 3). Structural recovery was calculated as the ratio of viscosity at the end of interval 3 to that at the end of interval 1. All tests were performed at 25 °C, except for the temperature ramp oscillatory test.

### 2.7. Statistical Analyses

Data were presented as mean ± standard deviation after testing for normality distribution using the Anderson–Darling test (α = 0.05). Data were obtained from two independent replicates for each oleogel sample. The paired Student’s *t*-test test was used to determine significant differences between the oil loss, hardness, and adhesiveness of canola oil or soybean oil oleogels stored at 4 °C or 25 °C. One-way analysis of variance (ANOVA) followed by Tukey’s post hoc test was used to determine the significant differences in the hardness, adhesiveness, and oil loss of the optimized oleogels. Pearson correlation was used to investigate the relationship between response variables considering all oleogel samples included in the experimental designs (*n* = 68). Values with *p* < 0.05 were considered statistically significant. GraphPad Prism version 8.0.0 for Windows (GraphPad Software, San Diego, CA, USA) was used to perform all statistical analyses.

## 3. Results and Discussion

### 3.1. Model Fitting

Oil loss (Table 2 and Table 3), hardness (Table 4 and Table 5), and adhesiveness (Table 6 and Table 7) of chitosan-based oleogels prepared according to the experimental design were accurately fitted to quadratic models, regardless of oil type or storage temperature of the oleogels. Oil loss models (Table 3) reached R^2^ > 0.88 (*p*-value < 0.0001), while hardness (Table 4) and adhesiveness (Table 5) models (R^2^ > 0.91, *p*-value < 0.0001) showed similar precision, except for adhesiveness models of soybean oil oleogels stored at 4 °C or 25 °C, which achieved R^2^ > 0.72 (*p*-value < 0.0001). This suggests that these oleogel samples were more susceptible to natural variations in their composition or environmental conditions than the other modeled samples. Nevertheless, R^2^ values ~0.70 can be considered sufficient for complex systems, such as oleogels [15]. The close agreement between R^2^ and adjusted R^2^ values indicates the models’ accuracy for oil loss, hardness, and adhesiveness. Additionally, there were no significant differences between the experimental and predicted data (*p*-value > 0.1) for canola oil and soybean oil oleogel models, regardless of storage temperature.

#### 3.1.1. Oil Loss

At 4 °C, oleogels exhibited a 10% reduction in oil loss (*p* < 0.0001) compared to storage at 25 °C. At 4 °C, canola oil oleogels demonstrated oil losses ranging from 16.8% to 42.7%, while soybean oil oleogels ranged from 22.1% to 41%. At 25 °C, oil losses increased to 26.4–53.2% for canola oil oleogels and 29.6–49.8% for soybean oil oleogels (Table 2). Higher storage temperatures increase molecular motion in oils, weakening hydrogen bonds within the oleogel structure and reducing oil viscosity and the gel’s oil-retention capacity [4,16]. Typically, oil losses below 10% are desired for food applications. However, the intermediate oil loss observed in most canola and soybean oil oleogels (<30%) could be advantageous for certain food applications requiring controlled oil release, such as flavor or nutrient delivery systems [9,17].

The type of oil did not significantly affect oil loss at 4 °C (*p* > 0.1). However, at 25 °C, oleogels with soybean oil showed 2.2% lower oil loss (*p* < 0.02) on average compared to those with canola oil (Table 2). Soybean oil contains 20% 18:1*n*-9, 52% 18:2*n*-6, and 5% 18:3*n*-3, giving it a higher degree of unsaturation than canola oil (60% 18:1*n*-9, 20% 18:2*n*-6, and 7% 18:3*n*-3) [18]. Oils with higher unsaturation are generally more polar, which strengthens hydrogen bonding with the polymer network at elevated temperatures, thereby enhancing oil retention [19].

Bascuas et al. [7] observed that flaxseed oil oleogels structured with hydroxypropylmethylcellulose and xanthan gum had 2–4% higher oil loss than those with olive or sunflower oils, despite flaxseed oil’s higher unsaturation. Conversely, Sivakanthan et al. [20] reported no differences in oil loss in oleogels structured with beeswax and stearic acid containing either rice bran or sesame oil, despite their distinct unsaturation levels. This inconsistency suggests that factors beyond oil unsaturation, such as fatty acid chain length and regiodistribution on triacylglycerols, minor compounds contents (e.g., mono- and diacylglycerols, free fatty acids, phenolics), and the type of organogelator, must also be considered [20,21,22].

The effects of chitosan, vanillin, and Tween^®^ 60 concentrations on the oil loss of oleogels are shown in Table 3. The best models obtained for canola oil oleogels stored at 4 °C and 25 °C are described in Equations (1) and (2). The models for soybean oil oleogels stored at 4 °C and 25 °C are described in Equations (3) and (4), where *x*_1_, *x*_2_, and *x*_3_ are the concentrations of chitosan, vanillin, and Tween^®^ 60, respectively.

(1) $$\mathrm{Oil}\;{\mathrm{loss}}_{\left(\mathrm{canola}\;\mathrm{oil}\;\mathrm{at}\;4\;{}^{\circ}C\right)}=27.3-8.60x_{1}\;+3.98x_{1}^{2}\;-3.02x_{3}^{2}\;-1.79x_{1}x_{3}$$

(2) $$\mathrm{Oil}\;{\mathrm{loss}}_{\left(\mathrm{canola}\;\mathrm{oil}\;\mathrm{at}\;25\;{}^{\circ}C\right)}=35.0-8.26x_{1}+5.41x_{1}^{2}+1.84x_{2}^{2}+2.87x_{3}-3.89x_{3}^{2}-1.02x_{1}x_{3}$$

(3) $$\mathrm{Oil}\;{\mathrm{loss}}_{\left(\mathrm{soybean}\;\mathrm{oil}\;\mathrm{at}\;4\;{}^{\circ}C\right)}=25.2-7.27x_{1}+6.93x_{1}^{2}-0.94x_{3}-1.24x_{1}x_{3}$$

(4) $$\mathrm{Oil}\;{\mathrm{loss}}_{\left(\mathrm{soybean}\;\mathrm{oil}\;\mathrm{at}\;25\;{}^{\circ}C\right)}=32.0-6.67x_{1}+4.87x_{1}^{2}-0.87x_{1}x_{3}+0.90x_{2}x_{3}$$

**Figure 1:**
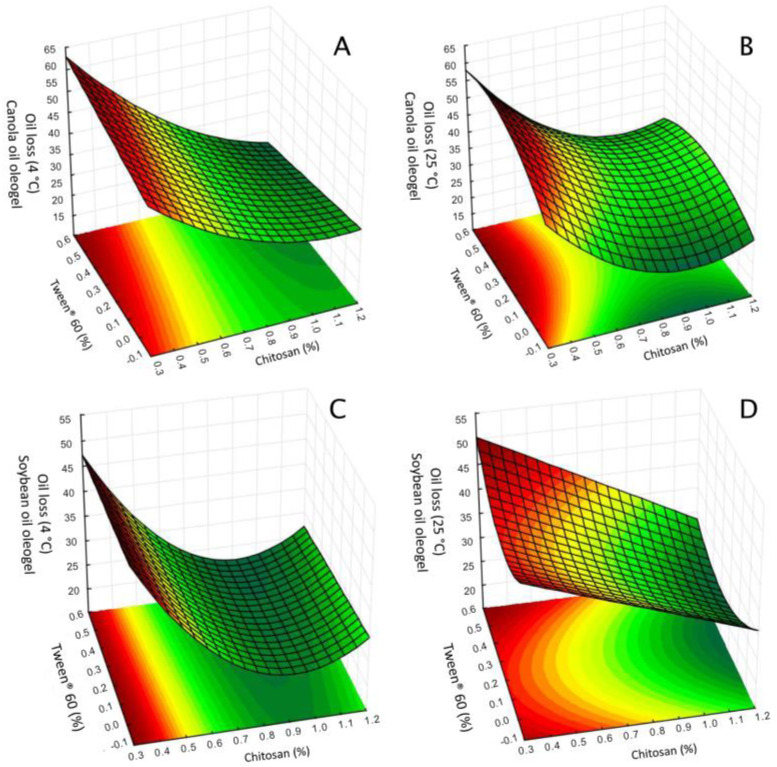
Response surface plot of the oil loss. Effect of the concentration of chitosan and Tween^®^ 60 with vanillin concentration fixed at central point. (**A**) Chitosan-based oleogels made with canola oil and stored at 4 °C for 24 h; (**B**) Chitosan-based oleogels made with canola oil and stored at 25 °C for 24 h; (**C**) Chitosan-based oleogels made with soybean oil and stored at 4 °C for 24 h; (**D**) Chitosan-based oleogel made with soybean oil and stored at 25 °C for 24 h.

The linear and quadratic terms of chitosan concentration were the most significant factors influencing oil loss in oleogels containing either canola or soybean oil, with both terms showing strong effects and low *p*-values (Table 3). The negative value of the linear term for chitosan suggests a reduction in oil loss with higher chitosan concentration. Conversely, the positive quadratic term for chitosan suggests that, after reaching a critical concentration (~1% chitosan), further increases in chitosan concentration yielded a smaller reduction in oil loss or even a slight increase (Figure 1).

Chitosan stabilizes the oleogel matrix by forming a network in which its amino groups (NH_2_^+^) are chemically modified through crosslinking with vanillin-derived aromatic aldehydes, resulting in Schiff bases. This crosslinking process mitigates electrostatic repulsion between chitosan chains and promotes rearrangement, facilitating hydrogen bonding between the hydroxyl groups (-OH) of vanillin and reactive groups on chitosan chains, thereby forming a robust three-dimensional network [6]. As chitosan concentration increases, more Schiff bases and hydrogen bonds form, thereby enhancing network density and stability and improving oil retention. Similar effects have been reported in chitosan-based oleogels containing olive oil [13] or camellia oil bodies [11,12], in which oil loss decreased with higher chitosan content. However, at higher concentrations, chitosan’s net positive charge may exacerbate electrostatic repulsion between polymer chains, disrupting the balance between polymer–polymer and polymer–oil interactions. This disruption can weaken the chitosan network’s ability to retain oil effectively [11]. Additionally, the interaction between chitosan and Tween^®^ 60 concentrations significantly reduced oil loss (Table 3).

Vanillin concentration did not affect oleogel oil loss, except in the case of canola oil oleogels at 25 °C, for which the quadratic term had a positive value (Table 3). This result was unexpected, given vanillin’s known role in facilitating crosslinked chitosan networks in oleogels, which in theory should enhance network density and reduce oil loss [11,12]. The limited effect of vanillin in this study may be due to the specific chitosan-to-vanillin ratio used (ranging from 2:5 to 21:1), which could have minimized vanillin’s influence on network formation. At these ratios, higher concentrations of chitosan might dominate the network structure, rendering the contribution of vanillin secondary or negligible in terms of oil retention.

This interpretation is supported by previous studies, such as those by Brito et al. [6] and Farooq et al. [11,12] which reported a notable effect of vanillin concentration on oil loss at lower chitosan-to-vanillin ratios (from 1:1 to 1:4). Under these conditions, a more balanced chitosan–vanillin interaction may facilitate stronger crosslinking, leading to reduced oil loss. Indeed, in our formulations, the lowest oil loss was observed for samples with a chitosan-to-vanillin ratio of approximately 1:1 (*w*/*w*) (Table 2). This finding suggests that the impact of vanillin on oil loss in oleogels is concentration-dependent, becoming more effective in promoting network stability when present in higher proportions relative to chitosan. In the current study’s conditions, elevated chitosan concentrations appear to overshadow vanillin’s effect, resulting in minimal contribution to oil retention. Therefore, adjusting the chitosan-to-vanillin ratio could optimize the oleogel network for improved stability at higher storage temperatures.

#### 3.1.2. Hardness

At 4 °C, oleogels exhibited higher hardness (*p* < 0.05) than those stored at 25 °C. At 4 °C, canola oil oleogels had hardness ranging from 4.31 N to 27.8 N, while soybean oil oleogels ranged from 4.81 N to 37.8 N. At 25 °C, hardness ranged from 2.94 N to 25.6 N for canola oil oleogels and from 4.67 N to 35.5 N for soybean oil oleogels (Table 4). Oil type significantly affected hardness (*p* < 0.05) at both temperatures, with soybean oil oleogels being consistently harder than those with canola oil. Furthermore, hardness was inversely correlated with oil loss (r = −0.72, *p* < 0.0001, *n* = 68).

Hardness is a key textural property for evaluating solid fats, as it indicates compressive strength and relates to the initial bite sensation during chewing. At lower temperatures, oleogels generally have higher viscosity and reduced molecular mobility, which lead to increased hardness [16]. Greater hardness typically reflects a more robust gel network with densely packed gelator molecules [23]. Oleogels containing oils with higher unsaturation levels tend to exhibit increased hardness because unsaturated carbon chains provide greater triglyceride flexibility, allowing for tighter packing within the polymer network. This enhanced packing strengthens polymer–polymer interactions, resulting in higher oleogel hardness [24,25]. Furthermore, oleogels with oils rich in saturated fatty acids show greater hardness compared to those with lower saturated fatty acids content [24,26,27]. Since soybean oil has a higher proportion of both polyunsaturated and saturated fatty acids than canola oil [18], soybean oil oleogels are expected to be firmer, as stronger gelator interactions and denser fatty acid packing enhance the structure.

The effects of the chitosan, vanillin, and Tween^®^ 60 concentrations on the hardness of oleogels are shown in Table 5. The best models for canola oil oleogels stored at 4 °C and 25 °C are given by Equations (5) and (6), respectively. Similarly, the models for soybean oil oleogels stored at 4 °C and 25 °C are shown in Equations (7) and (8), respectively, where x_1_, x_2_, and x_3_ are the concentrations of chitosan, vanillin, and Tween^®^ 60, respectively.

(5) $${\mathrm{Hardness}}_{\left(\mathrm{canola}\;\mathrm{oil}\;\mathrm{at}\;4\;{}^{\circ}C\right)}=14.0+8.28x_{1}+1.19x_{1}^{2}+2.04x_{2}+1.49x_{3}-1.56x_{3}^{2}+1.68x_{1}x_{2}+\ldots \;1.04x_{1}x_{3}-0.19x_{2}x_{3}$$

(6) $${\mathrm{Hardness}}_{\left(\mathrm{canola}\;\mathrm{oil}\;\mathrm{at}\;25\;{}^{\circ}C\right)}=9.92+5.77x_{1}+1.63x_{2}+1.56x_{3}+1.00x_{1}x_{2}+2.79x_{1}x_{3}+1.27x_{2}x_{3}$$

(7) $${\mathrm{Hardness}}_{\left(\mathrm{soybean}\;\mathrm{oil}\;\mathrm{at}\;4\;{}^{\circ}C\right)}=17.7+8.59x_{1}-1.78x_{1}^{2}+1.63x_{2}+5.37x_{3}+1.05x_{1}x_{2}+3.04x_{1}x_{3}+1.13x_{2}x_{3}$$

(8) $${\mathrm{Hardness}}_{\left(\mathrm{soybean}\;\mathrm{oil}\;\mathrm{at}\;25\;{}^{\circ}C\right)}=14.8+9.31x_{1}+1.50x_{1}^{2}+1.66x_{2}+1.21x_{2}^{2}+4.50x_{3}-1.85x_{3}^{2}+0.76x_{1}x_{2}+3.28x_{1}x_{3}$$

Regression analysis identified the linear term of chitosan concentration as the most significant factor influencing oleogel hardness, regardless of oil type or storage temperature (Table 5). This supports the notion that higher polymer concentrations enhance network strength, thereby increasing hardness [17,28,29]. Additionally, the linear terms of vanillin and Tween^®^ 60 concentrations and their interactions with chitosan (Table 5) significantly contributed to hardness, highlighting the synergistic importance of these ingredients in strengthening the chitosan network. Although quadratic terms for chitosan and Tween^®^ 60 concentrations, as well as the interaction between vanillin and Tween^®^ 60, also affected hardness, their impact was less substantial and not consistent across both oil types and storage temperatures.

Similar results have been observed in other studies, where increasing chitosan and vanillin concentrations in oleogels resulted in increased hardness [11,13]. Moreover, raising Tween^®^ 60 from 0% to 0.4% in canola oil oleogels structured with 0.75% chitosan doubled hardness, likely due to the formation of a more compact network [6]. In this study, the effects of vanillin and Tween^®^ 60 on hardness varied by oil type. In canola oil oleogels, vanillin concentration had a slightly greater impact on hardness, likely due to canola oil’s lower polarity, which may necessitate higher vanillin levels for effective network formation. In contrast, Tween^®^ 60 had a stronger effect in soybean oil oleogels. Tween^®^ 60, with an HLB value of approximately 14.6, is particularly effective at stabilizing emulsions with soybean oil, enhancing post-dehydration structural resistance [30].

While Tween^®^ 60 and vanillin had limited impact on oil loss (Table 3), their concentrations significantly influenced hardness (Table 5). Oil loss is primarily influenced by the network’s capacity to trap and immobilize the oil phase, which depends strongly on oil–structuring agent interactions. In contrast, hardness depends on the structural integrity and mechanical robustness of the network itself, which is influenced predominantly by the concentration and arrangement of the structuring agents within the gel [17]. Thus, different chemical determinants govern these two properties.

#### 3.1.3. Adhesiveness

Adhesiveness values ranged from 2.87 to 11.5 N·s^−1^ for canola oil oleogels and from 3.02 to 12.2 N·s^−1^ for soybean oil oleogels at 4 °C and from 1.98 to 10.8 N·s^−1^ and 2.70 to 10.4 N·s^−1^ at 25 °C, respectively (Table 6).

Adhesiveness refers to the force required to detach the oleogel surface from another surface, a property critical for predicting sensory texture perception and processing efficiency. High adhesiveness, for example, may reduce spreadability across surfaces [31]. The type of oil significantly affected adhesiveness only at 25 °C (*p* = 0.0004), with soybean oil oleogels being, on average, 1.5% more adhesive than those containing canola oil (Table 6). Additionally, increasing the storage temperature from 4 °C to 25 °C reduced the adhesiveness of canola oil oleogels by an average of 1.5% (*p* < 0.0001), though this temperature effect was not significant for oleogels containing soybean oil (*p* = 0.6605).

The effects of the chitosan, vanillin, and Tween^®^ 60 concentrations on the adhesiveness of chitosan-based oleogels are shown in Table 7. The best models for canola oil oleogels stored at 4 °C and 25 °C are given by Equations (9) and (10), respectively. The models for soybean oil oleogels stored at 4 °C and 25 °C are described in Equations (11) and (12), respectively, where x_1_, x_2_, and x_3_ are the concentrations of chitosan, vanillin, and Tween^®^ 60, respectively.

(9) $${\mathrm{Adhesiveness}}_{\left(\mathrm{canola}\;\mathrm{oil}\;\mathrm{at}\;4\;{}^{\circ}C\right)}=7.39+2.84x_{1}-1.40x_{1}^{2}+0.47x_{2}+0.69x_{2}^{2}+1.22x_{3}+0.65x_{1}x_{2}+0.55x_{1}x_{3}-0.53x_{2}x_{3}$$

(10) $${\mathrm{Adhesiveness}}_{\left(\mathrm{canola}\;\mathrm{oil}\;\mathrm{at}\;25\;{}^{\circ}C\right)}=6.44+2.34x_{1}-0.96x_{1}^{2}+0.72x_{2}+1.05x_{3}-0.72x_{3}^{2}+0.46x_{1}x_{2}+1.33x_{1}x_{3}$$

(11) $${\mathrm{Adhesiveness}}_{\left(\mathrm{soybean}\;\mathrm{oil}\;\mathrm{at}\;4\;{}^{\circ}C\right)}=8.24+1.77x_{1}-1.95x_{1}^{2}+1.31x_{3}$$

(12) $${\mathrm{Adhesiveness}}_{\left(\mathrm{soybean}\;\mathrm{oil}\;\mathrm{at}\;25\;{}^{\circ}C\right)}=7.70+1.93x_{1}+0.75x_{2}+1.50x_{3}-1.39x_{3}^{2}+0.86x_{1}x_{3}$$

According to regression coefficients and *p*-values, the linear effects of chitosan and Tween^®^ 60 concentrations were the most significant factors affecting the adhesiveness of oleogels regardless of temperature and oil type (*p* < 0.0001, Table 7). Increasing chitosan and Tween^®^ 60 concentrations generally increased adhesiveness, although the quadratic term of chitosan showed negative values, indicating diminishing returns at higher concentrations. Vanillin also contributed positively to adhesiveness (Table 7), supporting findings reported by Palla et al. [32], Pandolsook and Kupongsak [33], and Öǧütcü and Yilmaz [34], which showed that increasing concentrations of structuring agents (e.g., monoglycerides and natural waxes) lead to increased adhesiveness.

In this study, adhesiveness was directly correlated with hardness (r = 0.86, *p*-value < 0.0001, *n* = 68) and showed an inverse correlation with oil loss (r = −0.71, *p*-value < 0.0001, *n* = 68). Softer oleogels are typically more adhesive because their weaker gel structure allows for greater interaction with other surfaces [32,35]. However, in this study, chitosan and Tween^®^ 60 were found to contribute to a denser, more tightly packed network with increased structural strength (higher hardness), while also enhancing surface interactions, which increased the stickiness of the oleogels (higher adhesiveness).

Overall, the lowest oil loss and the highest hardness and adhesiveness were observed in the formulations containing 1.07% chitosan, 1.00% vanillin, and 0.5% Tween^®^ 60, with a chitosan-to-vanillin ratio approximately of 1:1 (*w*/*w*) (Table 2, Table 4, and Table 6). Oleogels with increased hardness and adhesiveness may be beneficial in applications requiring mechanical resistance, such as fillings or coatings. However, increased adhesiveness may pose handling challenges, necessitating a balance between adhesiveness, hardness, and oil retention to optimize performance in specific applications [2].

### 3.2. Model Validation and Multiobjective Optimization

The target values for oil loss were set based on the minimum observed values in oleogels containing canola oil (26.4%, Table 2) and soybean oil (29.6%, Table 2) at 25 °C. For hardness and adhesiveness, target values were established to match the mechanical properties of butter (10.8 N and 8.03 N·s^−1^), margarine from brand #1 (11.0 N and 8.05 N·s^−1^), margarine from brand #2 (34.7 N and 26.8 N·s^−1^), hydrogenated fat from brand #1 (26.4 N and 14.2 N·s^−1^), hydrogenated fat from brand #2 (60.0 N and 37.8 N·s^−1^), and palm fat (30.7 N and 19.6 N·s^−1^).

Soybean oil oleogels demonstrated greater effectiveness in mimicking solid fat properties, achieving higher global desirability scores (0.72–0.94, Table 8) compared to canola oil (0.63–0.88, Table 9). This contrast emphasizes the importance of oil type selection in chitosan-based oleogels. For both oils, the highest desirability values were observed in oleogels formulated to mimic the properties of butter and margarine from brand #1, indicating strong alignment with the target characteristics of these reference fats.

The most challenging solid fat to replicate was the partially hydrogenated fat from brand #2, which exhibited the lowest global desirability values for both soybean and canola oil oleogels (Table 8 and Table 9). This difficulty likely stems from its high industrial *trans* fatty acid content (37%, Table S1). The elevated proportion of *trans* fatty acids strengthens intermolecular interactions and crystallization density, leading to greater hardness and adhesiveness [36]—properties that are difficult to replicate in oleogels without compromising oil retention. Furthermore, the lower hardness observed in the partially hydrogenated fat from brand #1 (27% *trans* fatty acid) compared to brand #2’s underscores the direct relationship between *trans* fatty acids content, crystal structure, and mechanical properties. These findings emphasize the challenge of developing oleogels capable of mimicking the texture of highly structured solid fats rich in *trans* fatty acids.

All optimized canola oil oleogels were structured with high chitosan (>0.94%) and vanillin (1.0%) contents, while Tween^®^ 60 ranged from 0.19% to 0.5%. Higher Tween^®^ 60 contents were used to mimic solid fats with greater hardness and adhesiveness values, such as hydrogenated fat (brand #2), margarine (brand #2), and palm fat (Table 6). Similarly, all optimized soybean oil oleogels contained a high chitosan content (>0.99%) but exhibited variations in vanillin (0.24% to 1%) and Tween^®^ 60 (0.17% to 0.5%) contents. Increased concentrations of both components were required to replicate the properties of hydrogenated fat (brand #2), margarine (brand #2), and palm fat. In general, the optimized oleogels formulated with soybean or canola oils exhibited chitosan-to-vanillin ratios ranging from 1:1 to 4:1 (*w*/*w*) as the target values for hardness and adhesiveness did not necessarily correspond to their maximum values observed in the runs from the experimental design (Table 4 and Table 6).

**Figure 2:**
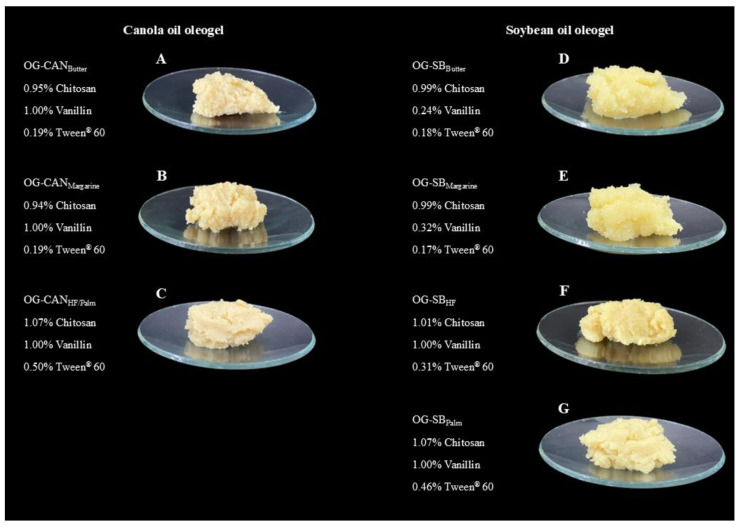
Visual appearance of optimized chitosan-based oleogels using canola oil (**A**–**C**) and soybean oil (**D**–**G**). (**A**) Canola oil oleogel mimetic of butter (OG-CAN_Butter_); (**B**) canola oil oleogel mimetic of margarine (OG-CAN_Margarine_; (**C**) canola oil oleogel mimetic of hydrogenated fat and palm fat (OG-CAN_HF/Palm_); (**D**) soybean oil oleogel mimetic of butter (OG-SB_Butter_); (**E**) soybean oil oleogel mimetic of margarine (OG-SB_Margarine_); (**F**) soybean oil oleogel mimetic of hydrogenated fat (OG-SB_HF_); (**G**) soybean oil oleogel mimetic of palm fat (OG-SB_Palm_).

All optimized oleogels demonstrated experimental response values that closely aligned with the predicted values, confirming the accuracy and reliability of the optimization process (Table 8 and Table 9). In particular, for oil loss, most responses had a residual standard error below 5%. Additionally, all model-predicted values fell within the confidence intervals of experimental results. For canola oil oleogels, the formulation containing 1.07% chitosan, 1.0% vanillin, and 0.5% Tween^®^ 60 was able to mimic margarine (brand #2), hydrogenated fat (brands #1 and #2), and palm fat, achieving desirability values above 0.63. In contrast, soybean oil oleogels required distinct formulations to mimic each solid fat. Optimized oleogels that exhibited a desirability value higher than 0.75 (canola oil) or 0.85 (soybean oil) were chosen for further investigation. The selected canola oil oleogels included those mimetics of butter (OG-CAN_Butter_), margarine brand #1 (OG-CAN_Margarine_); and hydrogenated fat brand #1 and palm fat (OG-CAN_HF/Palm_). For soybean oil oleogels, we chose those mimetics of butter (OG-SB_Butter_), margarine brand #1 (OG-SB_Margarine_), hydrogenated fat brand #1 (OG-SB_HF_), and palm fat (OG-SB_Palm_) (Figure 2).

### 3.3. Microstructure of Optimized Oleogels

**Figure 3:**
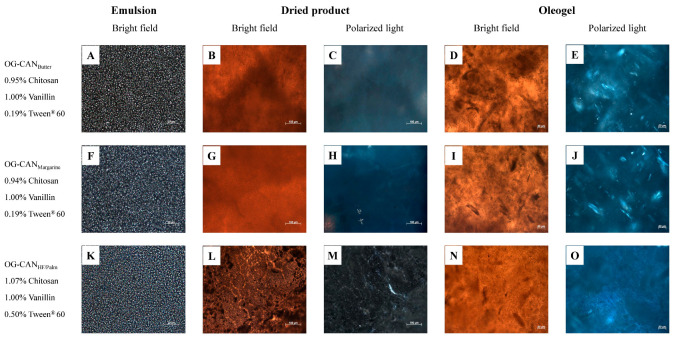
Microstructure of the optimized chitosan-based oleogels made with canola oil under bright-field and polarized light at 25 °C. Micrographs of emulsions (**A**,**F**,**K**), dried products ((**B**,**G**,**L**): bright field; (**C**,**H**,**M**): polarized light), and final oleogels ((**D**,**I**,**N**): bright field; (**E**,**J**,**O**): polarized light).

**Figure 4:**
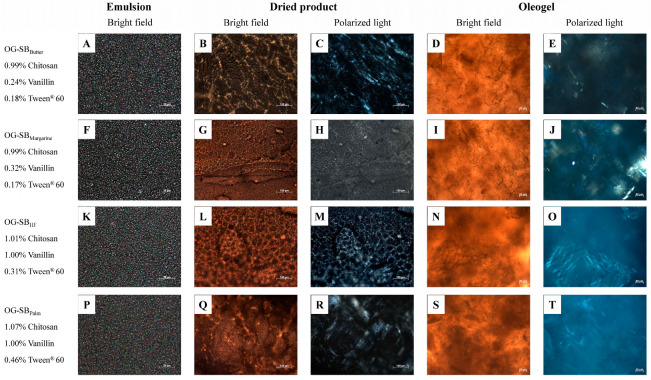
Microstructure of the optimized chitosan-based oleogels made with soybean oil under bright-field and polarized light at 25 °C. Micrographs of emulsions (**A**,**F**,**K**,**P**), dried products ((**B**,**G**,**L**,**Q**): bright field; (**C**,**H**,**M**,**R**): polarized light), and final oleogels ((**D**,**I**,**N**,**S**): bright field; (**E**,**J**,**O**,**T**): polarized light).

The production of chitosan-based oleogels by the emulsion-template method involves the formation of a stable oil-in-water emulsion followed by the removal of water through freeze-drying, resulting in tightly packed oil droplets within the chitosan network (dried product). This dried product is sheared to obtain the oleogel [6]. The microstructures of the selected optimized oleogels and their corresponding emulsions and dried products are shown in Figure 3 and Figure 4 for formulations containing canola and soybean oils, respectively. Bright-field microscopy reveals the chitosan network crosslinked with vanillin and highlights the role of Tween^®^ 60 in structuring. Polarized light micrographs detail vanillin crystals, emphasizing their contribution to the three-dimensional network.

Differences in the packing of oil droplets in emulsions were observed among the optimized formulations. The OG-CAN_HF/Palm_ emulsion (Figure 3K), with the highest content of chitosan and Tween^®^ 60, exhibited more tightly packed and uniformly distributed oil droplets compared to OG-CAN_Butter_ and OG-CAN_Margarine_ emulsions (Figure 3A,F). Likewise, OG-SB_HF_ and OG-SB_Palm_ emulsions (Figure 4K,P), with the highest content of chitosan, vanillin, and Tween^®^ 60, exhibited tighter oil droplet packing than OG-SB_Butter_ and OG-SB_Margarine_ emulsions (Figure 4A,F). This effect is attributed to the higher concentrations of Tween^®^ 60 (>0.31%), which reduce the interfacial tension between oil and water, forming smaller and more stable oil droplets during emulsification and leading to a more densely packed emulsion. Moreover, Tween^®^ 60 may increase the viscosity of the continuous phase, reducing the oil droplet mobility and maintaining a more uniform droplet distribution [37].

In the dried products, chitosan network reticulation varied across optimized formulations. OG-CAN_HF/Palm_, with higher chitosan content (1.07%), showed a denser, more interconnected, fibrous, and porous polymeric network structure (Figure 3L,M), forming oleogels with a continuous and compact network, without vanillin crystals (Figure 3N,O). The dried products of OG-CAN_Butter_ and OG-CAN_Margarine_ showed a less dense and reticulated network (Figure 3B,G), resulting in oleogels with visible vanillin crystals (Figure 3E,J), and reduced structural robustness, which aligns with their lower hardness.

The dried products of OG-SB_Palm_ (Figure 4Q,R) and OG-SB_HF_ (Figure 4L,M) were more robust and interconnected, forming oleogels with a more uniform and denser structure (Figure 4S,N) and fewer crystals of vanillin (Figure 4T,O). In fact, these oleogels were harder and showed lower oil loss. However, when OG-SB_Margarine_ and OG-SB_Butter_ emulsions were dried, aggregation of some emulsion droplets occurred, leading to the formation of structures with a lamellar pattern (Figure 4G,B) and loosely packed networks with discontinuous and thin fibers (Figure 4I,D) and resulting in softer oleogels with high oil losses. This outcome can be attributed to the lower vanillin content in the formulations, which resulted in a less crosslinked and structurally weaker oleogel.

These micrographs collectively demonstrated that higher concentrations of chitosan, vanillin and Tween^®^ 60 contribute to a denser and more stable microstructure. Brito et al. [6] observed a similar effect in their study on chitosan-based oleogels, in which crosslinking of chitosan with vanillin facilitated the formation of a densely interconnected network structure. Additionally, these authors also reported that the incorporation of Tween^®^ 60 reduced electrostatic repulsion between the polymeric chains, further enhancing the three-dimensional structure of the vanillin–chitosan network.

### 3.4. Rheological Behavior of Optimized Oleogels

#### 3.4.1. Oscillatory Amplitude Strain Sweep Test

All optimized oleogels showed a higher elastic modulus (G′) compared to the viscous modulus (G″), confirming their solid-like behavior (G′ > G″) within the linear viscoelastic region (Table 10). The predominance of elastic behavior suggests the formation of a more rigid gel, probably due to stronger intermolecular interactions, which contribute to a more entangled network. This network provides enhanced mechanical strength against external forces and greater resistance to elastic deformation [17].

**Figure 5:**
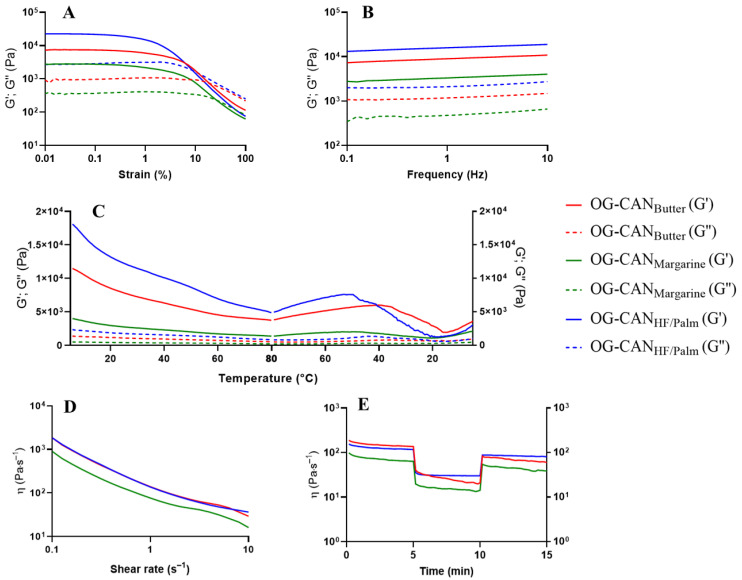
Rheological analysis of optimized formulation of canola oil oleogels: OG-CAN_Margarine_: 0.94% chitosan, 1.0% vanillin, 0.19% Tween^®^ 60 (green line); OG-CAN_Butter_: 0.95% chitosan, 1.0% vanillin, 0.19% Tween^®^ 60 (red line); OG-CAN_HF/Palm_: 1.07% chitosan, 1.0% vanillin; 0.50% Tween^®^ 60 (blue line). Elastic modulus (G′) is represented by the full lines and viscous modulus (G″) by the dotted lines. (**A**) Stress sweep test; (**B**) Frequency sweep test; (**C**) Oscillatory temperature test; (**D**) Shear-thinning behavior test; (**E**) Oscillatory shear behavior (recovery test).

Among the optimized canola oil oleogels, the increase in the concentrations of chitosan from 0.94% (OG-CAN_Margarine_) or 0.95% (OG-CAN_Butter_) to 1.07% (OG-CAN_HF/Palm_) and of Tween^®^ 60 from 0.19% (OG-CAN_Margarine_ and OG-CAN_Butter_) to 0.50% (OG-CAN_HF/Palm_) led to an enhancement in gel strength (increased G′) with an extension of the LVR and a higher force required to disintegrate the gel structure, as evidenced by the parameters of the critical oscillatory stress (σ critical) and yield stress (σ) (Table 10). These results suggest that higher concentrations of chitosan and Tween^®^ 60 contribute to a more robust gel network, making the oleogels more resistant to mechanical stress. Unexpectedly, the critical oscillatory strain (γ critical) did not follow the same behavior trend as σ critical. In this case, OG-CAN_Margarine_ and OG-CAN_Butter_ had high γ critical values (~0.25%), indicating that these oleogels can tolerate more deformation before failing compared to OG-CAN_HF/Palm_, which showed a low γ critical value (0.156%) (Table 10 and Figure 5A). The increased rigidity of OG-CAN_HF/Palm_ may have limited its flexibility, suggesting that, while the gel is mechanically stronger, it is more prone to failure under lower deformations [38]. Note that, although OG-CAN_Margarine_ and OG-CAN_Butter_ have nearly identical compositions, differing by only 0.01% chitosan concentration, their rheological properties differ significantly. This suggests that even minor variations in chitosan concentration can influence the local density distribution within the gel network, resulting in differences in their ability to withstand stress or deformation.

**Figure 6:**
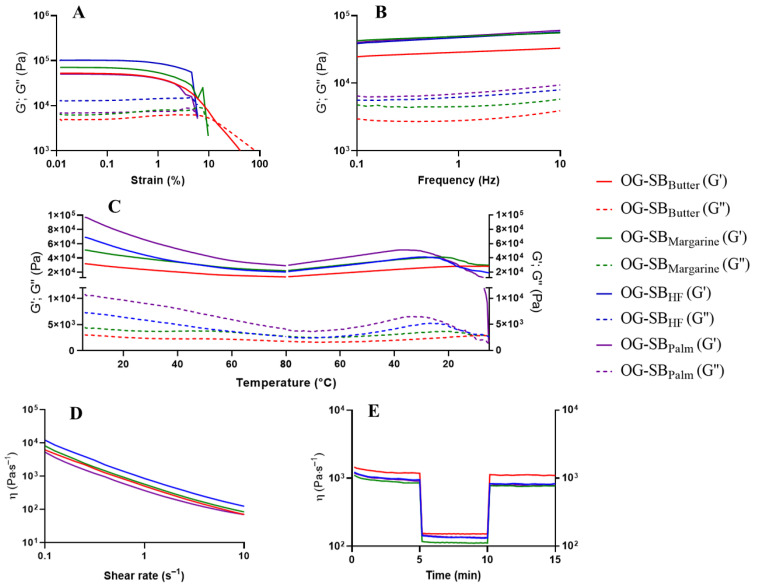
Rheological analysis of optimized formulation of soybean oil oleogels: OG-SB_Butter_: 0.99% chitosan, 0.24% vanillin, 0.18% Tween^®^ 60 (red line); OG-SB_Margarine_: 0.99% chitosan, 0.32% vanillin, 0.18% Tween^®^ 60 (green line); OG-SB_HF_: 1.01% chitosan, 1.0% vanillin, 0.31% Tween^®^ 60 (blue line); OG-SB_Palm_: 1.07% chitosan, 1.0% vanillin, 0.46% Tween^®^ 60 (purple line). Elastic modulus (G′) is represented by the solid lines and viscous modulus (G″) by the dotted lines. (**A**) Stress sweep test; (**B**) Frequency sweep test; (**C**) Oscillatory temperature test; (**D**) Shear-thinning behavior test; (**E**) Oscillatory shear behavior (recovery test).

Among optimized oleogels containing soybean oil, the increase in the concentration of chitosan from 0.99% (OG-SB_Margarine_ and OG-SB_Butter_) to 1.01% (OG-SB_HF_), vanillin from 0.24% to 1%, and Tween^®^ 60 from 0.18% to 0.31% also led to an enhancement in gel strength, as evidenced by an increase in all rheological parameters investigated, except for yield γ, in OG-SB_HF_ compared to OG-SB_Butter_ (Table 10 and Figure 6A). However, further increasing chitosan and Tween^®^ 60 concentrations from 1.01% to 1.07% and from 0.31% to 0.46%, respectively, negatively affected the formation of a more stable network in OG-SB_Palm_, resulting in lower structural strength and increased fragility, as demonstrated by the lower values of G′, σ critical, γ critical, σ yield, and γ yield compared to OG-SB_HF_ (Table 10). Therefore, it appears that there is a maximum concentration limit for chitosan and Tween^®^ 60 to optimally contribute to the formation of a robust gel network through intermolecular interactions. The formulation containing 1.01% chitosan, 1.00% vanillin, and 0.31% Tween^®^ 60 (OG-SB_HF_) promotes a more balanced interaction among the structuring agents, which may facilitate stronger crosslinking and enhance the mechanical strength of the oleogel.

Overall, optimized soybean oil oleogels exhibited superior mechanical properties (higher G′, σ critical, and σ yield values) compared to canola oil oleogels, indicating greater suitability for applications requiring enhanced structural integrity.

#### 3.4.2. Frequency Sweep Test

The frequency sweep graphs revealed that optimized oleogels containing canola (Figure 5B) and soybean oils (Figure 6B) had a weak dependence of G′ and G″ on frequency changes, showing characteristics of stronger gels, as previously observed for chitosan-based oleogels [6,11]. Additionally, G′ remained higher than G″ over the entire frequency range, confirming the structural stability of the gel. Optimized oleogels containing soybean oil showed higher G′ values than those containing canola oil, demonstrating their greater resistance to deformation due to their stronger elastic structure.

Despite this, optimized oleogels showed a constant loss of tangent (tan δ = G″/G′) across the frequency range, which was relatively low, with values ranging from 0.10 to 0.15. This indicates that G″ and G′ values remained proportionally balanced regardless of the frequency applied during the frequency sweep test. A constant tan δ value suggests that the structural integrity and viscoelastic properties of the oleogels are stable over a range of frequencies, indicating a robust gel network [29]. Additionally, the relatively low values of tan δ, below 1, indicate that the material’s elastic properties dominate over its viscous ones. This characteristic is especially important when considering oleogels as substitutes for solid fats in food systems, as they need to maintain structural stability without liquefying or deforming under dynamic conditions such as mixing or storage [1].

Regarding optimized oleogels containing canola oil (Figure 5B), higher G′ values were observed at increasing concentrations of chitosan and Tween^®^ 60, demonstrating the strongest elastic structure for OG-CAN_HF/Palm_, followed by OG-CAN_Butter_ and OG-CAN_Margarine_. For optimized oleogels containing soybean oil (Figure 6B), the effect of the concentrations of chitosan and Tween^®^ 60 on gel strength was less evident, since OG-SB_HF_, OG-SB_Margarine_, and OG-SB_Palm_ showed similar G′. However, it is noteworthy that OG-SB_butter_, which contains the lowest content of vanillin (0.24%), was the weakest of these oleogels.

#### 3.4.3. Temperature Ramp Test

Thermally reversible gelling behaviors of optimized oleogels during heating and cooling cycles are shown in Figure 5C and Figure 6C, respectively. The predominantly solid-like behavior (G′ > G″) of optimized oleogels remained consistent during the heating and cooling cycles, indicating thermal stability during temperature changes. The conditions selected for this rheological test allowed us to investigate the stability of the three-dimensional network of the oleogels across a broad temperature range. These included refrigeration temperature (5 °C), room temperature (25 °C), and elevated temperature conditions near to the melting point of pure vanillin, approximately 80 °C [39], where the interactions among chitosan, vanillin, and Tween^®^ 60 are expected to be weakest. All optimized oleogels showed a certain degree of structural loss near to 80 °C, however, the structured network of the soybean oil oleogels (Figure 6C) was able to maintain greater mechanical integrity than that of the canola oil oleogels (Figure 5C), suggesting superior thermal stability.

For the optimized oleogels containing canola oil, G′ tends to decrease as the temperature increases, particularly in OG-CAN_HF/Palm_ and OG-CAN_Butter_, which contain higher concentrations of chitosan (1.07% and 0.95%, respectively) compared to OG-CAN_Margarine_ (0.94%). Although the increase in chitosan concentration between OG-CAN_Butter_ (0.95%) and OG-CAN_Margarine_ (0.94%) is minimal, the reduction in G′ value was quite pronounced. Conversely, the negative impact of increasing Tween^®^ 60 on the network’s fragility during heating was evident when the G′ decrease was more pronounced for OG-CAN_HF/Palm_ (0.50% Tween^®^ 60) compared to OG-CAN_Butter_ (0.19% Tween^®^ 60). During cooling from 80 °C to 40 °C, all optimized oleogels containing canola oil exhibited an increase in G′, which was more evident in OG-CAN_HF/Palm_, followed by OG-CAN_Butter_ compared to OG-CAN_Margarine_ (Figure 5C). However, as cooling continued from 40 °C to 20 °C, G′ decreased again, and this cycle appeared to restart during further cooling from 20 °C to 5 °C. This phenomenon may be attributed to the ability of the compounds’ network in OG-CAN_HF/Palm_ and OG-CAN_Butter_ to reestablish interactions, bringing the polymer chains into closer proximity, leading to a partial restructuring of the network after a significant destabilization [6]. However, these newly formed networks seem to be weaker than the initial one, losing their ability to maintain the rearrangement of polymer chains over time, even with lower energy input.

Regarding optimized oleogels containing soybean oil (Figure 6C), G′ tends to decrease as the temperature increases from 5 °C to 80 °C in OG-SB_Palm_ and OG-SB_HF_, which contain the highest content of chitosan (1.07%), vanillin (1.00%), and Tween^®^ 60 (0.46%). In contrast, G′ of OG-SB_Margarine_ and OG-SB_Butter_, which contain the lowest content of structuring agents, remained constant during the heating cycle. During cooling from 80 °C to 5 °C, G′ of oleogels containing soybean oil remained unchanged, except for OG-SBPalm which showed a crossover point (G′ = G″) at low temperatures (~5 °C). High thermal stability of oleogels structured by chitosan crosslinked with vanillin containing canola [6] and soybean oil [9] has also been reported.

#### 3.4.4. Shear-Thinning Behavior and Structure Recovery Properties

The pseudoplastic behavior of optimized canola oil (Figure 5D) and soybean oil oleogels (Figure 6D) was confirmed by a significant decrease in apparent viscosity with increasing shear rate (1 to 100 s^−1^). This shear-thinning behavior likely resulted from the disruption of weak chemical interactions among the components of the three-dimensional network [6,9].

For optimized canola oil oleogels (Figure 5D), OG-CAN_HF/Palm_ and OG-CAN_Butter_ exhibited higher apparent viscosity than OG-CAN_Margarine_. A slight increase in chitosan concentration (from 0.94% to 0.95%), enhanced the apparent viscosity of the oleogels, possibly due to increased Schiff base formation [11]. In contrast, increasing Tween^®^ 60 concentration from 0.19% (OG-CAN_Butter_) to 0.50% (OG-CAN_HF/Palm_) did not significantly affect apparent viscosity. This higher Tween^®^ 60 concentration may have disrupted the polymeric network structuring, reducing the initial viscosity of the oleogels [6]. Nevertheless, the higher chitosan and Tween^®^ 60 levels in OG-CAN_HF/Palm_ improved structural recovery (69.2%) compared to OG-CAN_Margarine_ (60.8%) and OG-CAN_Butter_ (44.1%) (Figure 5E).

Considering optimized soybean oil oleogels, OG-SB_Palm_, with the highest concentrations of structuring agents, showed apparent viscosity values similar to OG-SB_Butter_ and OG-SB_Margarine_, which contained the lowest concentrations of these agents. Structural recovery followed the order OG-SB_Butter_ (92.9%) > OG-SB_Margarine_ (90.5%) > OG-SB_HF_ (87.3%) > OG-SB_Palm_ (85.1%) (Figure 5E). The high content of structuring agents compromised the stability of the three-dimensional network of OG-SB_Palm_ (Figure 6D). Although the increase in chitosan and vanillin concentrations increased the potential crosslinking points for the polymeric chain through covalent bonds (Schiff bases) and favored the development of intermolecular interactions, resulting in firmer oleogels, the higher emulsifier concentration may have favored the formation of weaker bonds, such as hydrogen bonds, thus making the oleogels more fragile and shear-sensitive [6,11].

## 4. Conclusions

Chitosan-based oleogels crosslinked with vanillin demonstrated their potential as substitutes for solid fats, particularly those containing soybean oil. Chitosan concentration was identified as the primary determinant of oil loss, hardness, and adhesiveness, with higher concentrations enhancing the structural integrity of the oleogels. Vanillin played a significant role in these properties in canola oil oleogels, while Tween^®^ 60 did so for soybean oil oleogels. Overall, oleogels containing a chitosan-to-vanillin ratio of approximately 1:1 (*w*/*w*) showed the lowest oil loss and the highest hardness and adhesiveness. Soybean oil oleogels achieved superior textural properties, mimicking butter and margarine with high desirability scores while maintaining intermediate oil loss. The optimized soybean oil oleogels exhibited enhanced gel strength, elasticity, and microstructural stability compared to the optimized canola oil oleogels, highlighting the influence of oil type on oleogel performance. Therefore, soybean oil oleogels provide a versatile alternative for solid fat replacement in food applications, contributing to the reduction of *trans* and saturated fats in the diet.

## Figures and Tables

**Table 1:** Coded and uncoded factors and levels of the experimental design.

Runs ^1^	Independent Variables					
	Chitosan		Vanillin		Tween^®^ 60	
1	−1	0.42%	−1	0.05%	−1	0.00%
2	−1	0.42%	−1	0.05%	+1	0.50%
3	−1	0.42%	+1	1.00%	−1	0.00%
4	−1	0.42%	+1	1.00%	+1	0.50%
5	+1	1.07%	−1	0.05%	−1	0.00%
6	+1	1.07%	−1	0.05%	+1	0.50%
7	+1	1.07%	+1	1.00%	−1	0.00%
8	+1	1.07%	+1	1.00%	+1	0.50%
9	−1	0.42%	0	0.52%	0	0.25%
10	+1	1.07%	0	0.52%	0	0.25%
11	0	0.75%	−1	0.05%	0	0.25%
12	0	0.75%	+1	1.00%	0	0.25%
13	0	0.75%	0	0.52%	−1	0.00%
14	0	0.75%	0	0.52%	+1	0.50%
15 (C) ^2^	0	0.75%	0	0.52%	0	0.25%
16 (C) ^2^	0	0.75%	0	0.52%	0	0.25%
17 (C) ^2^	0	0.75%	0	0.52%	0	0.25%

^1^ All runs were performed in a random order defined by the software. ^2^ Central point.

**Table 2:** Oil loss for chitosan-based oleogels with canola or soybean oils stored at 4 °C or 25 °C for 24 h.

Runs	Independent Variables			Oil Loss (%) ^1^			
	Chitosan (%)	Vanillin (%)	Tween^®^ 60 (%)	4 °C		25 °C	
				Canola	Soybean	Canola	Soybean
1	0.42	0.05	0.00	37.9 ± 3.58	39.0 ± 1.34	44.0 ± 0.00	43.3 ± 1.71
2	0.42	0.05	0.50	38.5 ± 0.38	37.7 ± 1.96	48.7 ± 0.58	42.5 ± 2.05
3	0.42	1.00	0.00	30.6 ± 0.65	38.5 ± 0.69	39.4 ± 0.83	40.4 ± 0.61
4	0.42	1.00	0.50	37.7 ± 0.72	40.9 ± 2.04	53.0 ± 0.23	48.3 ± 2.14
5	1.07	0.05	0.00	22.0 ± 0.97	28.1 ± 1.41	26.4 ± 1.62	30.2 ± 0.71
6	1.07	0.05	0.50	21.2 ± 1.78	24.0 ± 0.64	36.0 ± 2.17	30.9 ± 0.04
7	1.07	1.00	0.00	22.6 ± 0.41	26.7 ± 1.18	30.3 ± 1.75	30.3 ± 0.66
8	1.07	1.00	0.50	16.8 ± 1.86	22.1 ± 0.27	30.8 ± 2.63	29.6 ± 1.07
9	0.42	0.52	0.25	42.7 ± 1.16	41.0 ± 0.88	50.2 ± 1.35	43.1 ± 2.58
10	1.07	0.52	0.25	18.7 ± 0.08	23.5 ± 2.09	29.3 ± 1.69	29.9 ± 0.66
11	0.75	0.05	0.25	26.0 ± 1.00	29.1 ± 0.19	37.6 ± 0.12	35.7 ± 1.33
12	0.75	1.00	0.25	28.7 ± 1.91	24.8 ± 1.81	34.7 ± 0.82	30.8 ± 0.88
13	0.75	0.52	0.00	22.0 ± 0.82	26.8 ± 2.22	30.3 ± 1.22	34.5 ± 0.80
14	0.75	0.52	0.50	25.4 ± 0.51	24.9 ± 1.12	30.5 ± 1.29	30.7 ± 0.04
15	0.75	0.52	0.25	24.3 ± 0.87	23.1 ± 1.71	35.3 ± 3.05	30.3 ± 2.36
16	0.75	0.52	0.25	30.0 ± 2.38	25.2 ± 2.31	35.2 ± 0.75	31.0 ± 0.12
17	0.75	0.52	0.25	28.7 ± 1.99	22.6 ± 0.04	37.5 ± 0.29	31.0 ± 0.40
Mean				27.9	29.3	37.0	34.8

^1^ Results are expressed as mean ± standard deviation of two individual replicates for each run.

**Table 3:** Analysis of variance (ANOVA) for fitted quadratic polynomial models of oil loss (%) of chitosan-based oleogels with canola or soybean oils stored at 4 °C and 25 °C.

Variables	Oil Loss			
	4 °C		25 °C	
Oleogels Containing Canola Oil	Effect	*p*-Value	Effect	*p*-Value
Chitosan (L)	−17.2	0.0000	−16.5	0.0000
Chitosan (Q)	7.96	0.0001	10.8	0.0000
Vanillin (Q)	-	-	3.68	0.0111
Tween^®^ 60 (L)	-	-	5.74	0.0000
Tween^®^ 60 (Q)	−6.05	0.0014	−7.78	0.0000
Chitosan (L) x Tween^®^ 60 (L)	−3.58	0.0019	−2.04	0.0144
R^2^	0.88		0.89	
R^2^ adjusted	0.86		0.87	
*p*-value of model	0.0000		0.0000	
Oleogels containing soybean oil	Effect	*p*-value	Effect	*p*-value
Chitosan (L)	−14.5	0.0000	−13.3	0.0000
Chitosan (Q)	13.9	0.0000	9.74	0.0000
Tween^®^ 60 (L)	−1.88	0.0129	-	-
Chitosan (L) × Tween^®^ 60 (L)	−2.47	0.0044	−1.74	0.0130
Vanillin (L) × Tween^®^ 60 (L)	-	-	1.80	0.0105
R^2^	0.93		0.90	
R^2^ adjusted	0.92		0.88	
*p*-value of model	0.0000		0.0000	

**Table 4:** Hardness of chitosan-based oleogels with canola or soybean oil stored at 4 °C or 25 °C.

Runs	Independent Variables			Hardness (N)			
	Chitosan (%)	Vanillin (%)	Tween^®^ 60 (%)	4 °C		25 °C	
				Canola	Soybean	Canola	Soybean
1	0.42	0.05	0.00	4.31 ± 0.01	4.81 ± 0.26	4.16 ± 0.00	4.67 ± 0.12
2	0.42	0.05	0.50	6.44 ± 0.42	10.4 ± 0.81	2.94 ± 0.46	7.19 ± 0.44
3	0.42	1.00	0.00	6.92 ± 0.23	7.48 ± 0.88	6.38 ± 1.09	6.59 ± 0.54
4	0.42	1.00	0.50	5.63 ± 0.01	8.29 ± 1.06	3.37 ± 0.05	7.15 ± 0.64
5	1.07	0.05	0.00	14.4 ± 0.51	17.9 ± 2.19	11.2 ± 1.36	16.9 ± 1.59
6	1.07	0.05	0.50	19.7 ± 0.23	26.4 ± 5.37	14.3 ± 0.27	29.7 ± 1.58
7	1.07	1.00	0.00	22.7 ± 0.78	15.5 ± 0.66	10.6 ± 0.05	19.0 ± 0.10
8	1.07	1.00	0.50	26.7 ± 0.20	37.8 ± 1.47	25.6 ± 3.53	35.5 ± 2.35
9	0.42	0.52	0.25	5.22 ± 0.21	5.88 ± 0.09	3.71 ± 0.18	6.93 ± 0.64
10	1.07	0.52	0.25	27.8 ± 0.82	25.2 ± 0.28	16.6 ± 2.34	24.5 ± 2.28
11	0.75	0.05	0.25	13.4 ± 0.40	14.4 ± 0.57	8.02 ± 1.57	12.1 ± 1.16
12	0.75	1.00	0.25	16.7 ± 1.33	21.2 ± 1.53	11.1 ± 0.11	18.8 ± 0.75
13	0.75	0.52	0.00	11.3 ± 0.28	7.39 ± 0.24	9.70 ± 0.85	6.03 ± 0.02
14	0.75	0.52	0.50	16.2 ± 2.00	23.9 ± 2.40	11.4 ± 0.02	18.7 ± 0.91
15	0.75	0.52	0.25	13.3 ± 0.76	20.8 ± 1.62	9.20 ± 0.85	16.0 ± 0.39
16	0.75	0.52	0.25	13.2 ± 0.16	18.4 ± 1.22	10.7 ± 0.71	16.2 ± 0.73
17	0.75	0.52	0.25	10.9 ± 0.89	18.1 ± 0.42	9.68 ± 1.00	14.8 ± 1.00
Mean				13.8	16.7	9.92	15.3

Results are expressed as mean ± standard deviation of two individual replicates for each run.

**Table 5:** Analysis of variance (ANOVA) for fitted quadratic polynomial models of hardness of chitosan-based oleogels made with canola oil or soybean oil stored at 4 °C or 25 °C.

Variables	Hardness			
	4 °C		25 °C	
Oleogel containing canola oil	Effect	*p*-value	Effect	*p*-value
Chitosan (L)	16.6	0.0000	11.5	0.0000
Chitosan (Q)	2.39	0.0057	-	-
Vanillin (L)	4.08	0.0000	3.27	0.0000
Vanillin (Q)	-	-	-	-
Tween^®^ 60 (L)	2.99	0.0000	3.12	0.0000
Tween^®^ 60 (Q)	−3.12	0.0006	-	-
Chitosan (L) × Vanillin (L)	3.37	0.0000	2.00	0.0043
Chitosan (L) × Tween^®^ 60 (L)	2.08	0.0003	5.58	0.0000
Vanillin (L) × Tween^®^ 60 (L)	−1.18	0.0216	2.54	0.0006
R^2^	0.94		0.92	
R^2^ adjusted	0.92		0.90	
*p*-value of model	0.0000		0.0000	

**Table 6:** Adhesiveness values for chitosan-based oleogels containing canola oil or soybean oil and stored at 4 °C or 25 °C for 24 h.

Runs	Independent Variables			Adhesiveness (N·s^−1^)			
	Chitosan (%)	Vanillin (%)	Tween^®^ 60 (%)	4 °C		25 °C	
				Canola	Soybean	Canola	Soybean
1	0.42	0.05	0.00	2.87 ± 0.14	3.02 ± 0.16	2.68 ± 0.15	3.48 ± 1.10
2	0.42	0.05	0.50	5.12 ± 0.26	5.56 ± 0.46	1.98 ± 0.14	4.48 ± 0.97
3	0.42	1.00	0.00	3.96 ± 0.11	4.66 ± 0.34	3.46 ± 0.00	4.85 ± 1.95
4	0.42	1.00	0.50	3.34 ± 0.20	4.50 ± 0.61	2.26 ± 0.25	5.11 ± 0.18
5	1.07	0.05	0.00	6.28 ± 0.37	7.41 ± 0.85	4.02 ± 0.51	5.04 ± 1.12
6	1.07	0.05	0.50	9.99 ± 0.49	8.36 ± 0.18	7.68 ± 0.05	10.4 ± 2.60
7	1.07	1.00	0.00	9.24 ± 0.08	5.86 ± 0.40	5.65 ± 0.41	8.01 ± 0.00
8	1.07	1.00	0.50	11.5 ± 0.68	10.0 ± 0.02	10.8 ± 1.44	10.8 ± 1.15
9	0.42	0.52	0.25	3.25 ± 0.02	4.84 ± 0.48	2.47 ± 0.03	4.82 ± 0.39
10	1.07	0.52	0.25	9.86 ± 0.05	8.60 ± 0.22	8.08 ± 0.84	7.79 ± 0.60
11	0.75	0.05	0.25	8.20 ± 0.47	7.76 ± 0.81	5.14 ± 0.05	6.54 ± 1.29
12	0.75	1.00	0.25	9.08 ± 0.59	8.66 ± 0.50	6.55 ± 0.62	8.68 ± 0.12
13	0.75	0.52	0.00	5.10 ± 0.28	6.63 ± 0.17	3.69 ± 0.12	2.70 ± 0.30
14	0.75	0.52	0.50	9.65 ± 1.44	12.2 ± 1.76	7.34 ± 0.75	8.26 ± 0.77
15	0.75	0.52	0.25	6.72 ± 0.40	8.49 ± 0.73	6.68 ± 0.19	10.4 ± 0.31
16	0.75	0.52	0.25	7.61 ± 0.10	6.39 ± 0.45	6.51 ± 0.38	8.57 ± 1.23
17	0.75	0.52	0.25	6.76 ± 0.56	7.55 ± 0.40	7.74 ± 0.30	7.16 ± 0.21
Mean				7.00	7.10	5.45	6.90

Results are expressed as mean ± standard deviation of two individual replicates for each run.

**Table 7:** Analysis of variance (ANOVA) for fitted quadratic polynomial models of adhesiveness of chitosan-based oleogels made with canola oil or soybean oil stored at 4 °C or 25 °C.

Variables	Adhesiveness			
	4 °C		25 °C	
Oleogel containing canola oil	Effect	*p*-value	Effect	*p*-value
Chitosan (L)	5.67	0.0000	4.67	0.0000
Chitosan (Q)	−2.80	0.0000	−1.92	0.0006
Vanillin (L)	0.94	0.0007	1.44	0.0000
Vanillin (Q)	1.38	0.0043	-	-
Tween^®^ 60 (L)	2.44	0.0000	2.11	0.0000
Tween^®^ 60 (Q)	-	-	−1.43	0.0065
Chitosan (L) × Vanillin (L)	1.30	0.0001	0.91	0.0050
Chitosan (L) × Tween^®^ 60 (L)	1.09	0.0005	2.67	0.0000
Vanillin (L) × Tween^®^ 60 (L)	−1.07	0.0006	-	-
R^2^	0.93		0.93	
R^2^ adjusted	0.91		0.91	
*p*-value of model	0.0000		0.0000	

**Table 8:** Predicted and experimental responses of optimized oleogels containing soybean oil compared to commercial solid fats.

Targeted Solid Fat	Butter	Margarine		Partially Hydrogenated Fat		Palm Fat
		Brand #1	Brand #2	Brand #1	Brand #2	
Optimized formulation	0.99% Chitosan 0.24% Vanillin 0.18% Tween	0.99% Chitosan 0.32% Vanillin 0.17% Tween	1.07% Chitosan 1.00% Vanillin 0.50% Tween	1.01% Chitosan 1.00% Vanillin 0.31% Tween	1.07% Chitosan 1.00% Vanillin 0.50% Tween	1.07% Chitosan 1.00% Vanillin 0.46% Tween
	Oil loss (%)					
Target ^1^	28.7	28.7	28.7	28.7	28.7	28.7
Predicted	28.6 ± 1.27	28.7 ± 1.21	28.5 ± 1.93	27.5 ± 1.46	28.5 ± 1.93	27.8 ± 1.73
Observed	30.6 ± 0.59	31.1 ± 0.07	29.6 ± 1.07	27.5 ± 2.00	29.6 ± 1.07	26.7 ± 0.48
RSE (%)	+6.8	+8.5	+3.7	−0.2	+3.7	+3.8
	Hardness (N)					
Target ^2^	10.8	11.0	34.7	26.4	60.0	30.7
Predicted	19.7 ± 1.12	19.7 ± 1.07	33.9 ± 1.70	26.4 ± 1.29	33.9 ± 1.70	32.8 ± 1.52
Observed	20.1 ± 0.37	19.5 ± 1.37	35.5 ± 2.35	31.0 ± 4.53	35.5 ± 2.35	30.6 ± 6.30
RSE (%)	+2.2	−0.9	+4.7	+17.5	+4.7	−6.7
	Adhesiveness (N·s^−1^)					
Target ^2^	8.03	8.05	26.8	14.2	37.8	19.6
Predicted	8.03 ± 1.25	8.05 ± 1.20	11.3 ± 1.91	10.4 ± 1.45	11.3 ± 1.91	11.4 ± 1.71
Observed	10.1 ± 1.55	9.35 ± 0.62	10.8 ± 1.15	11.9 ± 1.24	10.8 ± 1.15	13.0 ± 0.99
RSE (%)	+25.7	+16.1	−4.8	+14.0	−4.8	+14.3
Global desirability	0.94	0.94	0.83	0.89	0.72	0.87

Results are expressed as mean ± standard deviation of independent triplicates for each oleogel. ^1^ The target for oil loss was determined based on the minimum value identified as the response in the experimental design. ^2^ Values obtained experimentally through the analysis of commercial fats. RSE (%): Residual standard error = ((Experimental value − Predicted value)/Predicted value × 100).

**Table 9:** Predicted and experimental responses of optimized oleogels containing canola oil compared to commercial solid fats.

Targeted Solid Fat	Butter	Margarine		Partially Hydrogenated Fat		Palm Fat
		Brand #1	Brand #2	Brand #1	Brand #2	
Optimized formulation	0.95% Chitosan 1.00% Vanillin 0.19% Tween	0.94% Chitosan 1.00% Vanillin 0.19% Tween	1.07% Chitosan 1.00% Vanillin 0.50% Tween	1.07% Chitosan 1.00% Vanillin 0.50% Tween	1.07% Chitosan 1.00% Vanillin 0.50% Tween	1.07% Chitosan 1.00% Vanillin 0.50% Tween
	Oil loss (%)					
Target ^1^	25.2	25.2	25.2	25.2	25.2	25.2
Predicted	31.3 ± 1.81	31.4 ± 1.79	31.2 ± 2.59	31.2 ± 2.59	31.2 ± 2.59	31.2 ± 2.59
Observed	30.9 ± 0.41	30.6 ± 0.85	30.8 ± 2.63	30.8 ± 2.63	30.8 ± 2.63	30.8 ± 2.63
RSE (%)	−1.3	−2.6	+1.2	+1.2	+1.2	+1.2
	Hardness (N)					
Target ^2^	10.8	11.0	34.7	26.4	60.0	30.7
Predicted	14.9 ± 1.47	14.8 ± 1.46	22.7 ± 2.10	22.7 ± 2.10	22.7 ± 2.10	22.7 ± 2.10
Observed	16.2 ± 1.59	14.7 ± 4.91	25.6 ± 3.53	25.6 ± 3.53	25.6 ± 3.53	25.6 ± 3.53
RSE (%)	+8.8	−0.2	+12.9	+12.9	+12.9	+12.9
	Adhesiveness (N·s^−1^)					
Target ^2^	8.03	8.05	26.8	14.2	37.8	19.6
Predicted	8.03 ± 0.69	8.05 ± 0.68	10.7 ± 0.98	10.7 ± 0.98	10.7 ± 0.98	10.7 ± 0.98
Observed	8.73 ± 0.20	7.03 ± 0.34	10.8 ± 1.44	10.8 ± 1.44	10.8 ± 1.44	10.8 ± 1.44
RSE (%)	+8.8	−12.6	+1.3	+1.3	+1.3	+1.3
Global desirability	0.88	0.88	0.68	0.79	0.63	0.72

Results are expressed as mean ± standard deviation of independent triplicates for each oleogel. ^1^ The target for oil loss was determined based on the minimum value identified as the response in the experimental design. ^2^ Values obtained experimentally through the analysis of commercial fats. RSE (%): Residual standard error = ((Experimental value − Predicted value)/Predicted value × 100).

**Table 10:** Rheological properties of optimized oleogel formulations.

Optimized Formulations	Linear Viscoelastic Region				Crossover Point	
	G′ (Pa)	G″ (Pa)	σ_critical_ (Pa)	γ_critical_ (%)	Yield σ (Pa)	Yield γ (%)
Canola oil oleogels						
OG-CAN_Butter _ (0.95% chitosan, 1.00% vanillin, 0.19% Tween)	7481.8	951.5	17.9	0.251	197.3	18.7
OG-CAN_Margarine _ (0.94% chitosan, 1.00% vanillin, 0.19% Tween)	2790.0	364.9	6.7	0.252	85.0	26.3
OG-CAN_HF/Palm _ (1.07% chitosan, 1.00% vanillin, 0.50% Tween)	22,537.7	2738.9	33.2	0.156	221.7	8.8
Soybean oil oleogels						
OG-SB_Butter _ (0.99% chitosan, 0.24% vanillin, 0.18% Tween)	52,571.1	4922.7	119.4	0.238	841.2	13.6
OG-SB_Margarine _ (0.99% chitosan, 0.32% vanillin, 0.17% Tween)	70,921.9	6284.5	157.2	0.234	1326.7	15.0
OG-SB_HF _ (1.01% chitosan, 1.00% vanillin, 0.31% Tween)	102,712.6	12,872.0	351.3	0.357	2356.2	11.9
OG-SB_Palm _ (1.07% chitosan, 1.00% vanillin, 0.46% Tween)	50,298.7	6912.1	144.3	0.301	764.7	7.8

## Data Availability

The original contributions presented in this study are included in the article/Supplementary Materials. Further inquiries can be directed to the corresponding authors.

## Supplementary Materials

The following supporting information can be downloaded at: https://www.mdpi.com/article/10.3390/polym17111526/s1, Table S1: Lipid composition of oils and fats used in the present study according nutritional facts in labels; Figure S1: FTIR spectrum of chitosan showing characteristic bands at ~3356 cm^−1^ (O–H and N–H stretching), 1647 cm^−1^ (C=O stretching, amide I), 1570 cm^−1^ (N–H bending, amide II), 1150 cm^−1^ (C–O–C stretching), and 1024 cm^−1^ (C–O stretching).
