# Supplementary material for: Optimization and Characterization of Crosslinked Chitosan-Based Oleogels Based on Mechanical Properties of Conventional Solid Fats
Source: Polymers (Basel). 2025 May 29;17(11):1526. doi: 10.3390/polym17111526 (PMC12157033; doi:10.3390/polym17111526)
Supplement: Supplementary file 1 [file polymers-17-01526-s001.zip › polymers-3640227-supplementary.pdf]

Article

Optimization and Characterization of Crosslinked Chitosan-Based Oleogels Based on Mechanical Properties of Conventional Solid Fats

Supplementary Material

Supplementary Table S1: Lipid composition of oils and fats used in the present study according nutritional facts in labels

Product	Total Fat (%)	Saturated fatty acids (%)	Monounsaturated fatty acids (%)	Polyunsaturated fatty acids (%)	*Trans*-fatty acids (%)
Canola Oil	100	8.3	60.8	29.1	Do not contain
Soybean Oil	100	16.6	24.2	59.1	Do not contain
Butter	82	55	NI	NI	3*
Margarine brand #1 with interesterified vegetable oil	80	24	NI	NI	Do not contain
Margarine brand #2 with partially hydrogenated fat	70	30	NI	NI	0.7
Partially Hydrogenated Fat brand #1	100	27	NI	NI	27
Partially Hydrogenated Fat brand #2	100	25	NI	NI	37
Palm Fat	100	51	NI	NI	Do not contain

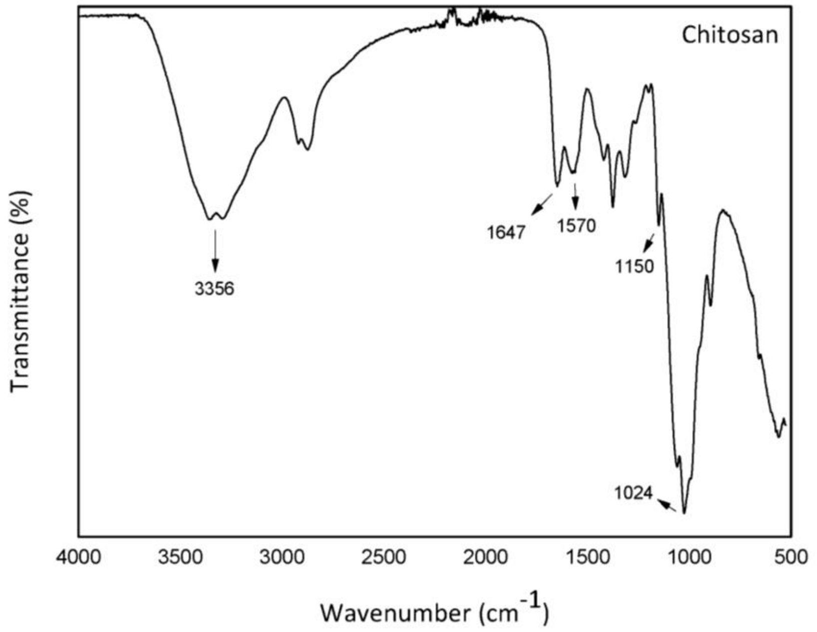

Supplementary Figure S1. FTIR spectrum of chitosan showing characteristic bands at ~3356 cm^{-1} (O–H and N–H stretching), 1647 cm^{-1} (C=O stretching, amide I), 1570 cm^{-1} (N–H bending, amide II), 1150 cm^{-1} (C–O–C stretching), and 1024 cm^{-1} (C–O stretching).
